# Porcine fibrin sealant combined with autologous chondrocytes successfully promotes full‐thickness cartilage regeneration in a rabbit model

**DOI:** 10.1002/term.3224

**Published:** 2021-06-04

**Authors:** Yu Yang, Xin Wang, Kangkang Zha, Zhuang Tian, Shuyun Liu, Xiang Sui, Zhigang Wang, Jilian Zheng, Jun Wang, Xiaobin Tian, Quanyi Guo, Jinmin Zhao

**Affiliations:** ^1^ Department of Orthopedics Trauma and Hand Surgery The First Affiliated Hospital of Guangxi Medical University Nanning Guangxi China; ^2^ Institute of Orthopedics The First Medical Center Chinese PLA General Hospital, Beijing Key Lab of Regenerative Medicine in Orthopedics, Key Laboratory of Musculoskeletal Trauma and War Injuries PLA Beijing China; ^3^ The Second People's Hospital of Guiyang Guiyang Guizhou China; ^4^ The Third Medical Center of PLA General Hospital Beijing China; ^5^ Department of Human Anatomy Histology and Embryology School of Basic Medical Sciences Peking University Beijing China; ^6^ Guizhou Medical University University Town Guiyang Guizhou China

**Keywords:** autologous chondrocytes (ACs), cartilage defect, cartilage repair, microfracture, porcine fibrin sealant (PFS), scaffold

## Abstract

Xenogeneic porcine fibrin sealant (PFS), derived from porcine blood, was used as a scaffold for cartilage tissue engineering. PFS has a porous microstructure, biocompatibility and degradation, and it provides a perfect extracellular matrix environment for the adhesion and proliferation of chondrocytes. Recently, PFS in combination with autologous chondrocytes (ACs) were used to study the microstructure of PFS scaffolds and promotion effect on the proliferation and migration of ACs. In this study, we investigated the effects of PFS in combination with ACs on the healing of cartilage defects in rabbits. A full‐thickness cartilage defect was made in the femoral trochlear in rabbits, subsequently, three surgical procedures were used to repair the defect, namely: the defect was treated with microfracture (MF group); the defect was filled with PFS alone (PFS group) or in combination with ACs (PFS + ACs group); the unrepaired cartilage defects served as the control group (CD group). Three and 6 months after the operation, the reparative effect was evaluated using medical imaging, gross scoring, pathological staining, biomechanical testing and biochemical examination. The PFS group showed a limited effect on defect repair, this result was significantly worse than the MF group. The best reparative effect was observed in the PFS + ACs group. These results hinted that PFS in combination with autologous chondrocytes has broad prospects for clinical applications in cartilage tissue engineering.

## 1 INTRODUCTION

Osteoarthritis (OA) is a prevalent musculoskeletal disease worldwide. Approximately 303 million people suffer greatly from OA (Kloppenburg & Berenbaum, 2020). OA affects any joint in the body, especially load‐bearing joints, such as the hip and knee. OA is the main cause of physical disability, and it places a significant burden on patients and the health care system, with great socioeconomic cost (Hunter & Bierma‐Zeinstra, 2019). It is possible to restore joint function and prevent the further development of OA in knee joints with cartilage damage by repairing the cartilage (Taheem et al., 2020). Articular cartilage is a highly hydrated viscoelastic connective tissue without innervation, vascularization, or lymphatic circulation. It contains a low density (accounting for 1%–5% of the total tissue volume) of chondrocytes with poor proliferative activity and a dense anti‐adhesion extracellular matrix (ECM) around the cells. Three type of cartilage regeneration techniques are used clinically, including microfracture (MF), autologous chondrocyte implantation (ACI), and osteochondral autogenous transplantation (OAT; Camarero‐Espinosa et al., 2016). However, these methods have numerous limitations and deficiencies. For example, MF leads to the formation of fibrocartilage with poor mechanical properties (Gao et al., 2018). The fixation of ACI is not firm, and there is a defect of a large number of cells lost (McCarthy et al., 2016), and the source of donor in OAT is limited, OAT has poor integration with surrounding cartilage (Nie et al., 2020).

Recently, tissue‐engineered cartilage shows incomparable advantages. There are generally two types of biomaterials used for cartilage tissue engineering, synthetic polymers produced by chemical reactions and natural polymers produced by living organisms. Compared to synthetic polymers (e.g., polylactide, polyglycolide, and polycaprolactone), natural polymers (e.g., fibrin, collagen, and chitosan) have many unique advantages, such as biocompatibility, biodegradability, porosity and adjustable mechanical properties (Bao et al., 2020). Fibrin is a natural polymer that provides a porous matrix to easily promotes cell adhesion, maintain cell vitality and promote cell proliferation (Whelan et al., 2014). The United States Food and Drug Administration (FDA) approved fibrin sealant (FS) for clinical use in 1998 (Roberts et al., 2020), and surgeons evaluated it as excellent. Most of the fibrinogen in commercial FS is extracted from human serum. Despite the availability of advanced virus detection technologies, the potential risk of viral disease transmission remains (Roberts et al., 2020).

Xenogeneic porcine fibrin sealant (PFS) was derived from porcine blood. Pigs have high homology with humans, and they exhibit similar anatomy, physiology and genetics. The safety performance of porcine plasma is greatly improved compared to cattle blood, snake venom and other animal sources. The risk of virus transmission is also greatly reduced. Now, PFS is widely used in clinical surgery for haemostasis. Therefore, in this study, we used xenogeneic PFS as a scaffold for cartilage tissue engineering to fill rabbit knee cartilage defects.

Chondrocytes as seed cells of tissue engineering cartilage have a higher chondrogenic capacity and exhibit greater secretion of growth factors and cytokines. The FDA approved autologous cell transplantation therapy (Colombini et al., 2019). And it has been widely used in tissue engineering. Then, the aim of the present study was to systematically evaluate the reparative effects of xenogeneic PFS in combination with or without autologous chondrocytes (ACs) on the healing of cartilage defects in rabbits using medical imaging, gross scoring, pathological staining, biomechanical testing and biochemical examination.

## 2 MATERIALS AND METHODS

### 2.1 Animals

Our experimental procedures were performed in compliance with the standards for the care and use of laboratory animals. 50 New Zealand white rabbits (5 months old, weighing 2.5−3.0 kg) were purchased from the Center of Animal Experiments and the experimental scheme was approved by the Animal Experiment Ethics Committee (Chinese People's Liberation Army General Hospital). A full‐thickness chondral defect was created in both knees of 30 rabbits. The left knee joint cartilage was collected from the other 20 rabbits to isolate and culture chondrocytes that were subsequently implanted into the defect in the right knee joint.

### 2.2 In vitro experiments

#### 2.2.1 Isolation and culture of autologous chondrocytes

**FIGURE 1:**
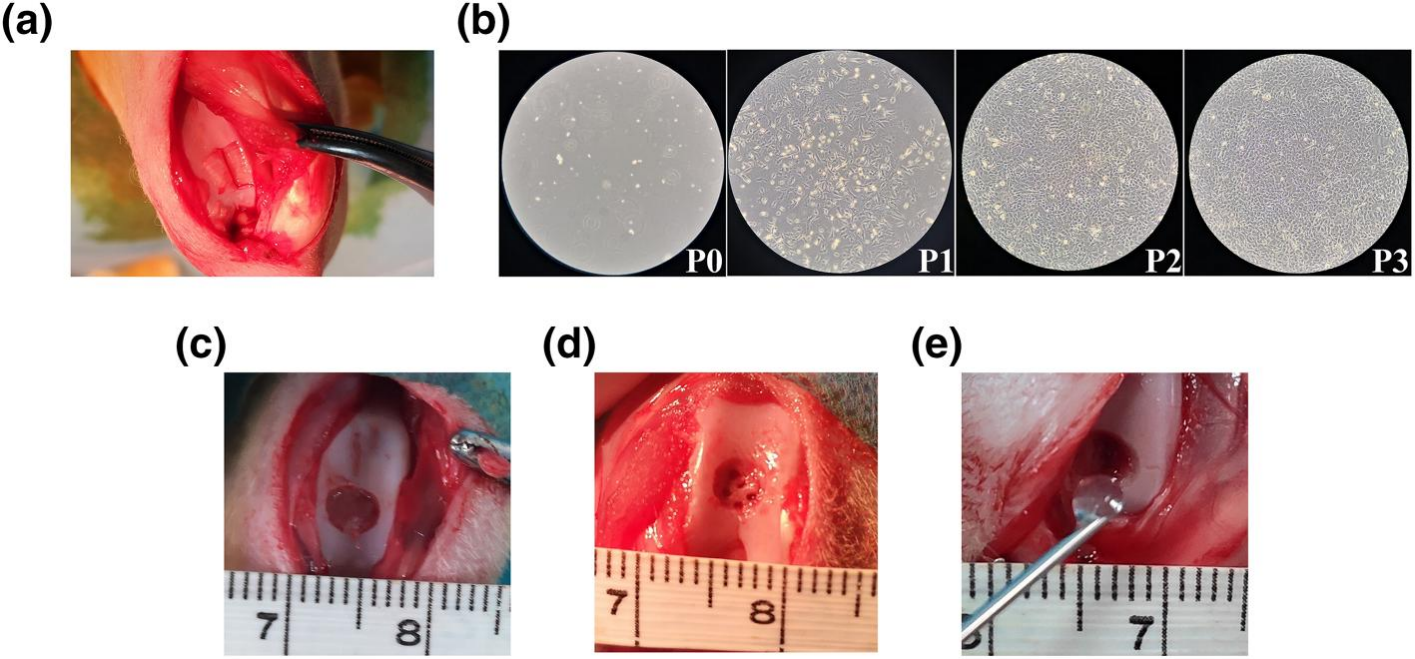
(a) Harvest of articular cartilage. (b) Morphological observation of P0‐P3 ACs by inverted microscopy (×40). (c) A 3.5‐mm‐diameter, 1‐mm‐deep, full‐thickness chondral defect was made in the trochlear groove. (d) The defect was treated with MF. (e) PFS + ACs were injected into the defect. ACs, autologous chondrocytes; MF, microfracture; PFS, porcine fibrin sealan.

Twenty rabbits were anaesthetized via an intramuscular injection of 10% xylazine hydrochloride (0.2 ml/kg, Huamu Animal Health Products Co., Ltd.), and the left knee joint cartilage was peeled away using a scalpel (Figure 1a). All rabbits were returned to the cage for the subsequent implantation of autologous chondrocytes (ACs) in the right knee joint.

Harvested articular cartilage was cut into 1 mm^3^ pieces and digested using 0.1% type II collagenase for 16 h. Acquired cells were cultured in Dulbecco's modified Eagle's medium/F‐12 Ham's (DMEM/F12) with 20% foetal bovine serum (FBS) and 1% penicillin–streptomycin then placed in an incubator (37°C, 5% CO_2_). The chondrocytes were seeded at 1 × 10^4^ cells/cm^2^, and the same density was used at each passage. Passage 3(P3) chondrocytes were used in the following experiments (Figure 1b).

#### 2.2.2 Characterization of porcine fibrin sealant

##### Preparation of porcine fibrin sealant

porcine fibrin sealant (PFS) was prepared according to the manufacturer's instructions (Hanbang Medical Technology Co., Ltd.). Briefly, freeze‐dried fibrinogen (fibrinogen 120 mg, coagulation XIII factor 20 IU) was dissolved in 2 ml of a sodium chloride (18 mg) solution (solution A), and lyophilized thrombin (1000 IU) was dissolved in 2 ml of a calcium chloride (11.76 mg) solution (solution B). These two solutions (A + B) were mixed at a 1:1 volume in a double‐barrelled syringe (with two plungers, a connecting Y‐piece and casing application) until a hydrogel‐like PFS was acquired after 2–4 min at room temperature.

##### Transwell chemotaxis assay

PFS was prepared by mixing solution A and solution D (mixture of 0.5 ml 0.9% NaCl and 0.1 ml of solution B) through a double‐barrelled syringe at equal volumes. PFS (0.2 ml) was injected into the lower chamber of the Transwell chamber system. After the PFS was solidified, 600 µl DMEM/F12 with 20% FBS was added to cover it. While the control group only contained 20% FBS DMEM in the lower chamber. Following incubation (37°C, 5% CO_2_) for 24 h, serum‐free DMEM/F12 (100 µl) with ACs concentration of 10^5^/ml was added to the upper chamber in each group and incubated again for 24 h. The non‐migratory cells in the upper chamber were removed with cotton swabs. Migratory cells that appeared on the lower side of the chamber were fixed with 4% paraformaldehyde for 30 min and stained with 0.1% crystal violet for 10 min. Five fields of vision at the center and in the surrounding areas in each sample were selected for quantification of the migrated cells using Image J (version 1.5.0, NIH). Three independent samples were evaluated in each group.

##### Structure of porcine fibrin sealant

Three PFS hydrogels were randomly prepared according to the above method. The configured PFS was fixed using 2.5% glutaraldehyde and 1% osmic acid tetroxide. Fixed PFS was subjected to gradient ethanol dehydration and critical point drying. PFS sections were fixed and sprayed with gold. The microscopic porous structure of PFS was observed using scanning electron microscopy (SEM, SU‐8100) at 3.0 kV. Three fields of vision in each PFS hydrogel were randomly selected to take pictures under the SEM.

#### 2.2.3 Combination of porcine fibrin sealant with autologous chondrocytes

PFS was combined with ACs by mixing solution A and solution C, which was acquired via the addition of 0.5 ml of a P3 AC suspension (2.5 × 10^7^/ml) to 0.1 ml of solution B, through a double‐barrelled syringe at equal volumes.

#### 2.2.4 Cell activity assay

A total of 0.2 ml of PFS + ACs, mentioned above, was cultured for 0, 7, and 14 days for the detection of live/dead cells. We stained the PFS + ACs complex (three independent samples were observed at each culture time) using the Live Death^@^ Viability/Cytotoxicity Kit (BioVision) according to the manufacturer's instructions. AC survival in PFS was observed using a confocal microscope (TCS‐SP8) at 488 and 552 nm. Three visual fields were randomly chosen under the confocal microscope for each sample to take pictures. Image J (version 1.5.0, NIH) was used to measure fluorescence intensity of living and dead cells in each field to calculate the percentage of dead cells.

#### 2.2.5 Cell proliferation detection

According to the instructions of Cell counting kit‐8 (CCK‐8, Dojindo Laboratory, Japan), we selected low concentration of chondrocytes for proliferation experiment. Briefly, we mixed 50 μl P3 AC suspension (1 × 10^5^/ml) with 10 μl solution B and added it to the 48‐well plate, followed by 60 μl solution A to the well plate. After the PFS solidified into a hydrogel, 600 μl of DMEM/F12 (with 20% FBS) was added to each well. The same number of cells in 600 μl of DMEM/F12 (with 20% FBS) were incubated as the blank control group (three independent samples were tested in each group at each time). The CCK‐8 assay (each sample had 3 vice‐holes) was used to estimate the cell proliferation rate within the PFS at days 0, 3, 5, and 7. “Vice‐holes” were defined as: pipetting 300 ul solution (10% CCK8 reagents in DMEM) of each sample from the 48‐well plate and distributing evenly into 3 holes of 96‐well plate (100 μl/hole) to measure the absorbance.

### 2.3 In vivo animal experiments

#### 2.3.1 Articular cartilage defect rabbit model

All rabbits were anesthetized via an intramuscular injection of 10% xylazine hydrochloride (0.2 ml/kg). The knee joints were disinfected, and the skin was incised to expose the femoral trochlea. A full‐thickness chondral defect (3.5 mm in diameter, 1 mm in depth) in the center of the trochlear groove was drilled without damaging the subchondral bone plate using a special punch (Figure 1c). The rabbits were divided into four groups: the defects in both knee joints of 10 rabbits were not treated (CD group); the defects in both knee joints of 10 rabbits were filled with 0.2 ml of PFS alone (PFS group); the defect in the both knee joints of 10 rabbits were treated with MF (the MF group); and the defects in the right knees of 20 rabbits was filled with 0.2 ml of a mixture of PFS and 1 × 10^7^/ml ACs originating from the rabbit's left knee joint (PFS + ACs group; Figure 1e). For the MF group, a 0.8‐mm hole was drilled into the subchondral bone, and bone marrow was introduced into the defect (Figure 1d). All animals were euthanized 3 and 6 months after surgery to collect knee joint samples for further evaluation.

#### 2.3.2 Medical imaging evaluation

##### Magnetic resonance imaging examination

A small‐animal Magnetic resonance imaging (MRI) system (BioSpec70/20USR) was used to evaluate the reparative effects on cartilage. The scanning sequence was T2, and the scanning parameters were set as described in a previous report (Lu et al., 2018). Three blinded independent musculoskeletal radiologists analysed all MRI images using the Whole‐Organ Magnetic Resonance Imaging Score (WORMS), which includes cartilage, marrow abnormality, and bone cysts (Peterfy et al., 2004).

##### Micro‐computed tomography (μ‐CT) analysis

After MRI, the repaired areas of the knee joints were exposed. The degree of subchondral bone repair in the knee joints was evaluated using a μ‐CT imaging system (EXPLORE LOCUS, GE Healthcare). The scanning parameters were set as previously reported (Lu et al., 2018). Axial, coronal, and sagittal images of the femoral condyle were reconstructed and analysed using GE Health Care MicroView ABA 2.1.2 software. A cylindrical (diameter, 3.5 mm; height, 1.5 mm) 3D region of interest (ROI) was established to ensure the inclusion of repaired tissue in the defect area in the ROI. Histomorphometric parameters of the subchondral bone, including the bone volume fraction (BVF), trabecular thickness (Tb.Th) and trabecular spacing (Tb.Sp), were calculated and analysed.

#### 2.3.3 Gross macroscopic assessment and scoring

Three blinded independent joint surgeons captured and evaluated macroscopic photos of the distal femur using the International Cartilage Repair Society (ICRS) scoring system after μ‐CT. This scoring system includes the degree of defect regeneration, integration of the defect edge, appearance of the surrounding cartilage, and an overall judgement (Peterson et al., 2000, van den Borne et al., 2007).

#### 2.3.4 Histological and immunohistochemical evaluation

After the ICRS score was determined, tissue samples from the femoral trochlea of rabbits in each group, including samples of cartilage in the repaired area, surrounding normal cartilage, underlying subchondral bone, and cancellous bone, were collected for further analyses. All samples were fixed in 4% paraformaldehyde, decalcified in 10% EDTA and dehydrated. The samples were embedded in paraffin and sliced into 5‐μm‐thick sections. General morphology was evaluated using haematoxylin and eosin (H&E) staining (Solarbio, G1121), and glycosaminoglycan (GAG) content was evaluated using safranin O/fast green staining (Solarbio, G1371). All staining methods were performed according to the instructions of the corresponding kits (HE staining kit, safranin O/fast staining kit). The expression of type II collagen was examined using immunohistochemical staining. Briefly, dropping 3% H_2_O_2_ on the sections after dewaxing and incubating for 30 min in room temperature avoiding light. Then adding 30 μl 0.25% pepsin (abcam, ab64201) on the samples followed by incubation in 37°C for 10 min to retrieve the antigen. After that, blocking tissue using 10% goat serum (Solarbio, SL038) for 30 min and incubating with Anti‐collagen II antibodies (Novus, NB600‐844) in 4°C overnight. Finally, samples were reacted with secondary antibody (MXB, KIT‐5020) for 1 h and colored by DAB (MXB, DAB‐0031) for 5 min. Three blinded independent pathologists evaluated images of the staining results using the ICRS II Visual Histological Assessment Scale (Mainil‐Varlet et al., 2010).

#### 2.3.5 Biomechanical testing

The repaired cartilage surface was placed perpendicular to the axis of indentation, and a cylindrical flat‐end indenter with a diameter of 1 mm (BOSE3220 EM USA) was used to continuously record the force and displacement applied to the repaired cartilage at a speed of 0.005 mm/s for 300 s. The elastic modulus of the repaired cartilage was calculated from the stress‐strain curve.

#### 2.3.6 Biochemical analyses

The regenerated tissue was removed from the knee defect using a corneal trephine for GAG and total collagen quantification according to the instructions of a GAG kit (GenMed Scientifics, Inc.) and hydroxyproline kit (Nanjing Jiancheng Bioengineering Institute), which was used to quantify the total collagen content.

### 2.4 Statistical analyses

All experimental data were statistically analysed using SPSS 23.0 software, and the values of the results are expressed as the means ± standard deviation. Date were analyzed by *t*‐test and One‐way analysis of variance (ANOVA) followed by LSD post hoc test. *p* < 0.05 was considered a significant difference.

## 3 RESULTS

### 3.1 Characterization of porcine fibrin sealan

**FIGURE 2:**
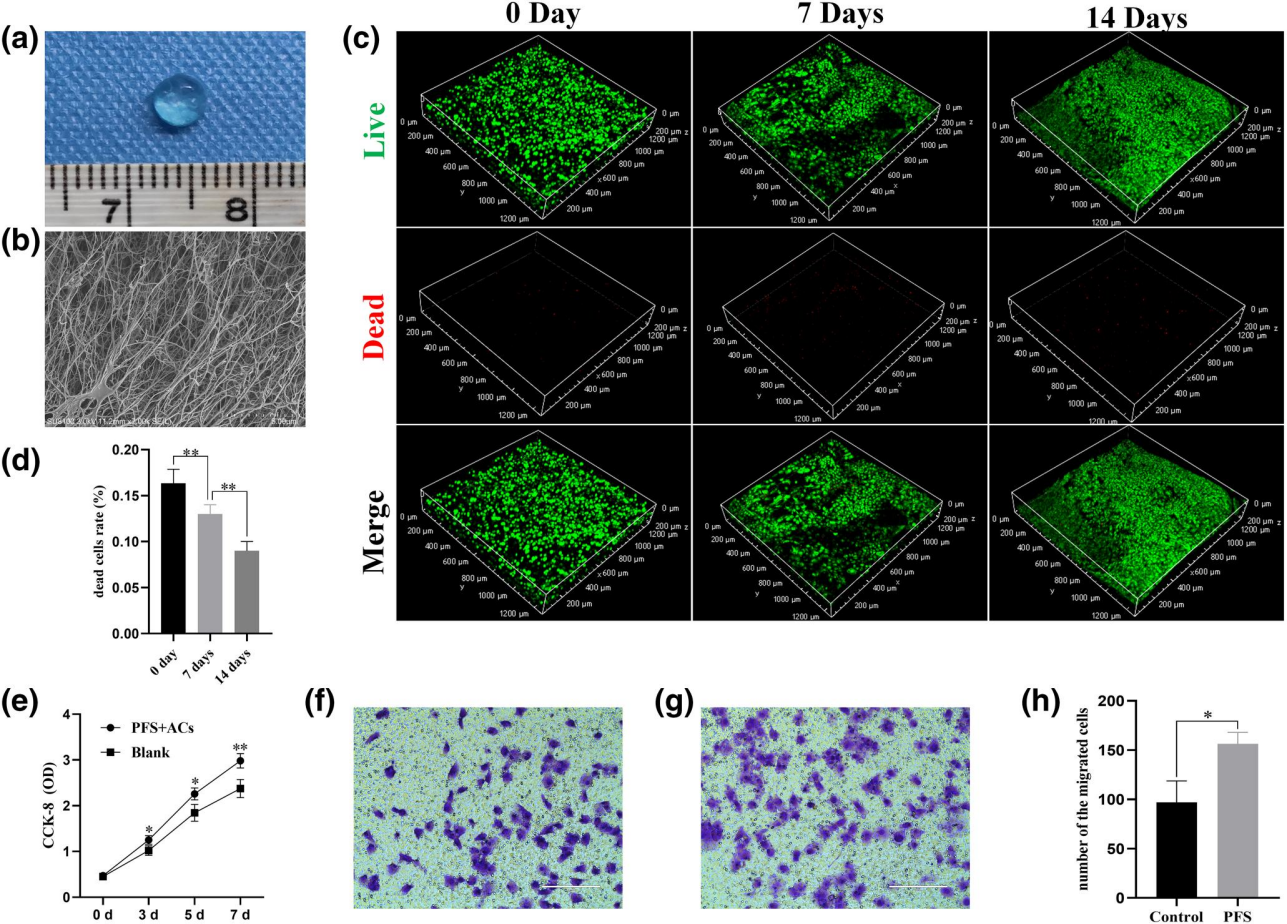
(a) Macroscopic observation of PFS. (b) SEM images of PFS (×7 K). (c) Live/dead cell staining of PFS + ACs hydrogel after 0, 7 and 14 days of culture. (d) dead cells rate of PFS + AC hydrogel after 0, 7 and 14 days of culture (*n* = 3, **p < 0.01). (e) CCK‐8 detection of PFS + ACs after 0, 3, 5 and 7 days of culture (*n* = 3, *p < 0.05, **p < 0.01). (f) Migration effect of DMEM/F12 with 20% FBS on ACs (bar: 200 μm). (g) Migration effect of DMEM/F12 with 20% FBS added PFS on ACs (bar: 200 μm). (h) number of the migrated cells in each group. The results are presented as the mean ± SD (*n* = 3, *p < 0.05). CCK, Cell counting kit; DMEM, Dulbecco's modified Eagle's medium/F‐12 Ham's; FBS, foetal bovine serum; PFS, porcine fibrin sealan; SEM, scanning electron microscopy

PFS is transparent and soft, but it has a certain toughness (Figure 2a). SEM revealed an interconnected porous structure in the interior of the PFS (Figure 2b). PFS + ACs were cultured for 0, 7 and 14 days followed by staining for live/dead cells. A large number of live cells (green fluorescence) and only a few dead cells (red fluorescence) were observed using confocal microscopy. A large number of ACs fused together with the increasing co‐culture time of PFS and ACs (Figure 2c). Moreover, with the extension of culture time, the proportion of dead cells decreased significantly (Figure 2d). The CCK‐8 assay (Figure 2e) showed that the absorbance values in both groups increased with increasing culture time, but the value in the PFS group increased faster than the blank control group (*p* < 0.05).

### 3.2 Migration effect of porcine fibrin sealan autologous chondrocytes

The migration effect of PFS (Figure 2g) on ACs was significantly stronger than the control group (Figure 2f), and there was a statistical difference (Figure 2h) between the two groups.

### 3.3 Magnetic resonance imaging evaluation of cartilage repair

**FIGURE 3:**
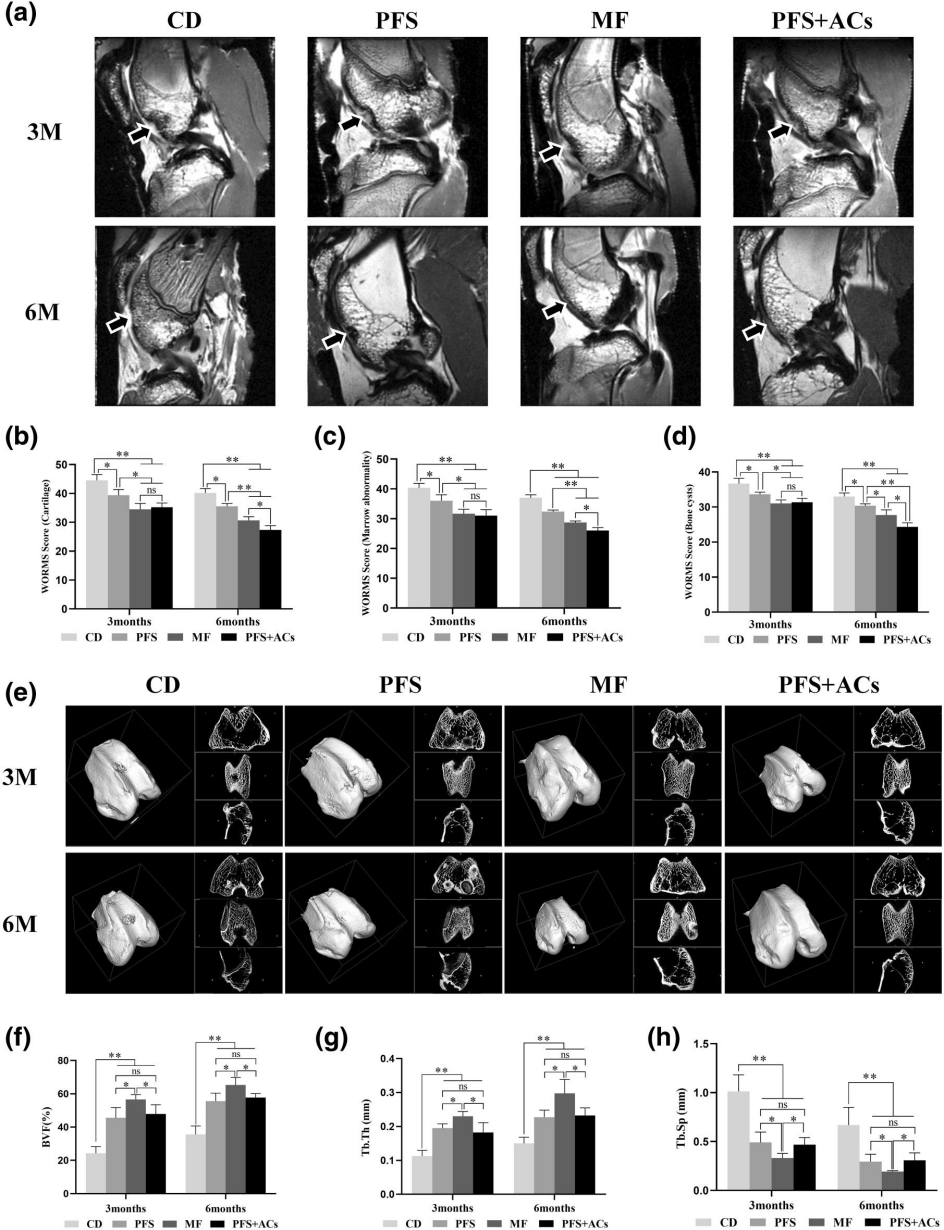
(a) MRI images from each group at each time point after the operation. (b–d) WORMS for cartilage (b), marrow abnormality (c), and bone cysts (d). The results of b–d are presented as the mean ± SD (*n* = 3, *p < 0.05, **p < 0.01). (e) μ‐CT images of each group at each time point after the operation. (f–h) Quantitative μ‐CT analysis of regenerated bone within the defect space in terms of the bone volume fraction (BVF) (f), trabecular thickness (Tb.Th) (g), and trabecular spacing (Tb.Sp) (h). The results of F‐H are presented as the mean ± SD (*n* = 4, *p < 0.05, **p < 0.01) MRI, Magnetic resonance imaging; WORMS, whole‐organ magnetic resonance imaging score

Three months after the operation (Figure 3a), the defect in the CD group was obvious, with an interrupted and sunken linear structure with a low signal on the defect surface and no tissue regeneration. The PFS, MF, and PFS + AC groups showed a low‐signal, interrupted linear structure and depression. Regenerated tissue, with medium and low signal intensities was seen in the PFS group, and nonuniform regenerated tissue with a medium signal intensity was observed in the MF and PFS + AC groups. The WORMS results showed that the cartilage (Figure 3b), marrow abnormality (Figure 3c) and bone cyst (Figure 3d) scores were highest in the CD group (*p* < 0.05). All scores in the PFS group were higher than the MF and PFS + ACs groups (*p* < 0.05). There were no statistical differences in the scores between the MF and PFS + ACs groups. A higher score here indicates a poorer effect.

Six months after the operation, the low‐signal linear structure in the defect area in the CD group remained unrecovered with no tissue regeneration. The defect area in the PFS group was significantly smaller than before. The defect was completely repaired in the MF and PFS + AC groups. The linear structure with a low signal intensity was restored, and a uniform layer of tissue with a medium signal intensity was observed on the surface of the defect. The WORMS in all groups decreased dramatically (*p* < 0.05) compared to 3 months after the operation. All scores in the CD, PFS, MF, and PFS + AC groups decreased in sequence with significant differences (*p* < 0.05), which means that the PFS + AC group exhibited the best reparative effect.

### 3.4 μ‐CT estimation of subchondral bone regeneration

Three months after the operation, μ‐CT images showed subchondral bone collapse and trabecular bone structure destruction in all knee joint cartilage defects (Figure 3e). The subchondral bone was seriously destroyed in the CD group, with a disordered trabecular bone structure and the worst BVF (Figure 3f), Tb.Th (Figure 3g), and Tb.Sp (Figure 3h) values of all groups (*p* < 0.01). Subchondral and trabecular bone growth was observed in the PFS and PFS + AC groups and did not exceed the surrounding normal subchondral bone. There were no significant differences in the values of BVF, Tb.Th or Tb.Sp between the PFS and PFS + AC groups. The proliferation of subchondral bone was especially obvious in the MF group, which showed growth that already exceeded the normal plane. The BVF and Tb.Th values in the MF group were higher the PFS and PFS + AC groups, and the Tb.Sp value was lower (*p* < 0.05).

Six months after the operation, the defect in the CD group remained obvious with little subchondral bone regeneration. The regenerated tissue in the PFS and PFS + AC groups fused with the surrounding normal subchondral bone, with a smooth defect surface and regular bone trabeculae. These two groups showed no significant differences (*p* > 0.05). The proliferation of subchondral bone in the MF group was more evident than at 3 months. BVF and Tb.Th were highest in the MF group, and the Tb.Sp was the lowest (*p* < 0.05), which corresponds to the earlier results.

### 3.5 Macroscopic observation of cartilage repair

**FIGURE 4:**
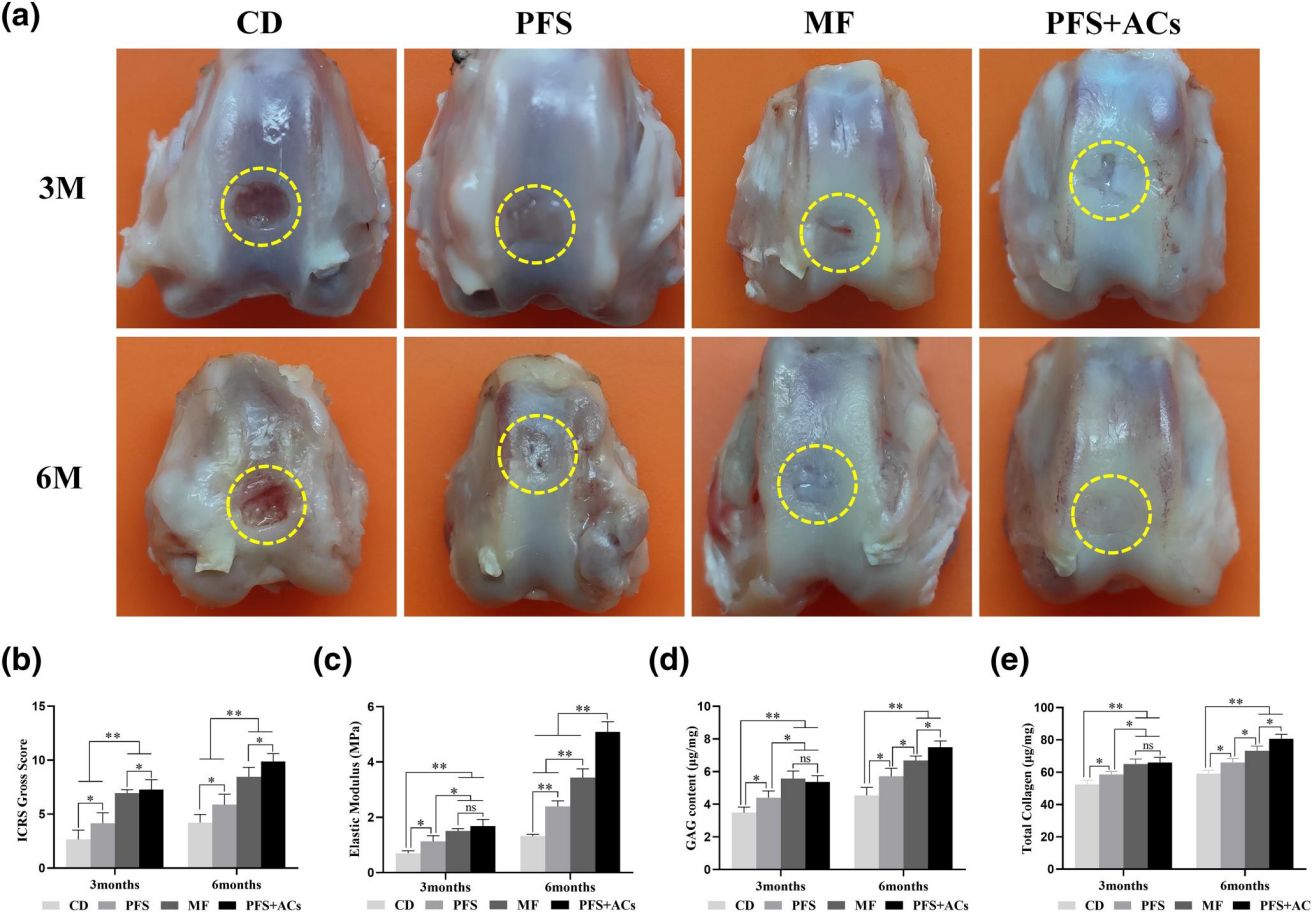
(a) Cross‐sectional appearance of regenerated cartilage in each group at each time point after the operation. (b) The ICRS gross score for each group. The results are presented as the mean ± SD (*n* = 5. *p < 0.05, **p < 0.01). () Young's modulus; () GAG content; (e) total collagen content. The results of c–e are presented as the mean ± SD (*n* = 3, *p < 0.05, **p < 0.01) GAG, glycosaminoglycan; ICRS, international cartilage repair society

Three and 6 months after the operation, gross observation of cartilage repair in the knee joints of rabbits in each group was performed (Figure 4a). Three months after surgery, the defect in the CD group was clearly visible, and there was almost no tissue regeneration. The defect in the PFS group was partially filled with regenerated tissue, which had a plane lower than the normal cartilage with a rough surface and a clear boundary between the new tissue and the normal cartilage tissue. Most of the defects in the MF and PFS + ACs groups were filled with regenerated tissue, with a smooth surface and blurred border. The ICRS scores (Figure 4b) in the CD and PFS groups were significantly lower than the other two groups (*p* < 0.01). The score in the CD group was lower (*p* < 0.05) than the PFS group, and the score in the PFS + ACs group was higher than the MF group.

Six months after the operation, the reparative effect in each group was greatly improved compared to 3 months. The defect in the CD group was clearly visible, with little tissue regeneration. The defect in the PFS group was mostly filled with new tissue with a plane lower than the normal cartilage. There were voids in the middle of the regenerated tissue and clear margins. The defect in the MF group was almost filled with regenerated tissue. The plane of the new tissue was slightly lower than the normal cartilage, with a smooth surface and blurred boundary. The defect in the PFS + ACs group was completely filled with transparent and smooth regenerated tissue, which integrated with the normal cartilage tissue. The scores in the PFS + ACs and MF groups were significantly higher than the other two groups (*p* < 0.01). The score in the CD group was lower (*p* < 0.05) than the PFS group, and the score in the PFS + ACs group was higher (*p* < 0.05) than the MF group.

### 3.6 Biomechanical analysis and biochemical components of regenerated cartilage

Analyses of the elastic modulus (Figure 4c), GAG content (Figure 4d) and total collagen content (Figure 4e) 3 months after the operation showed lower values for all 3 metrics in the CD group (*p* < 0.05) than the PFS group, and these values were significantly lower (*p* < 0.01) than those in the MF and PFS + AC groups. There was no significant difference (*p* > 0.05) between the MF and PFS + AC groups. Six months after the surgery, the elastic modulus, GAG and total collagen content were increased in all groups (*p* < 0.05). However, the differences in the elastic modulus, GAG and total collagen content between the CD group and the other groups were the same as 3 months after the operation. Notably, the elastic modulus, GAG and total collagen content increased more rapidly in the PFS + AC group than the MF group, and these values were highest in the PFS + AC group.

### 3.7 Histological evaluation of regenerated cartilage

**FIGURE 5:**
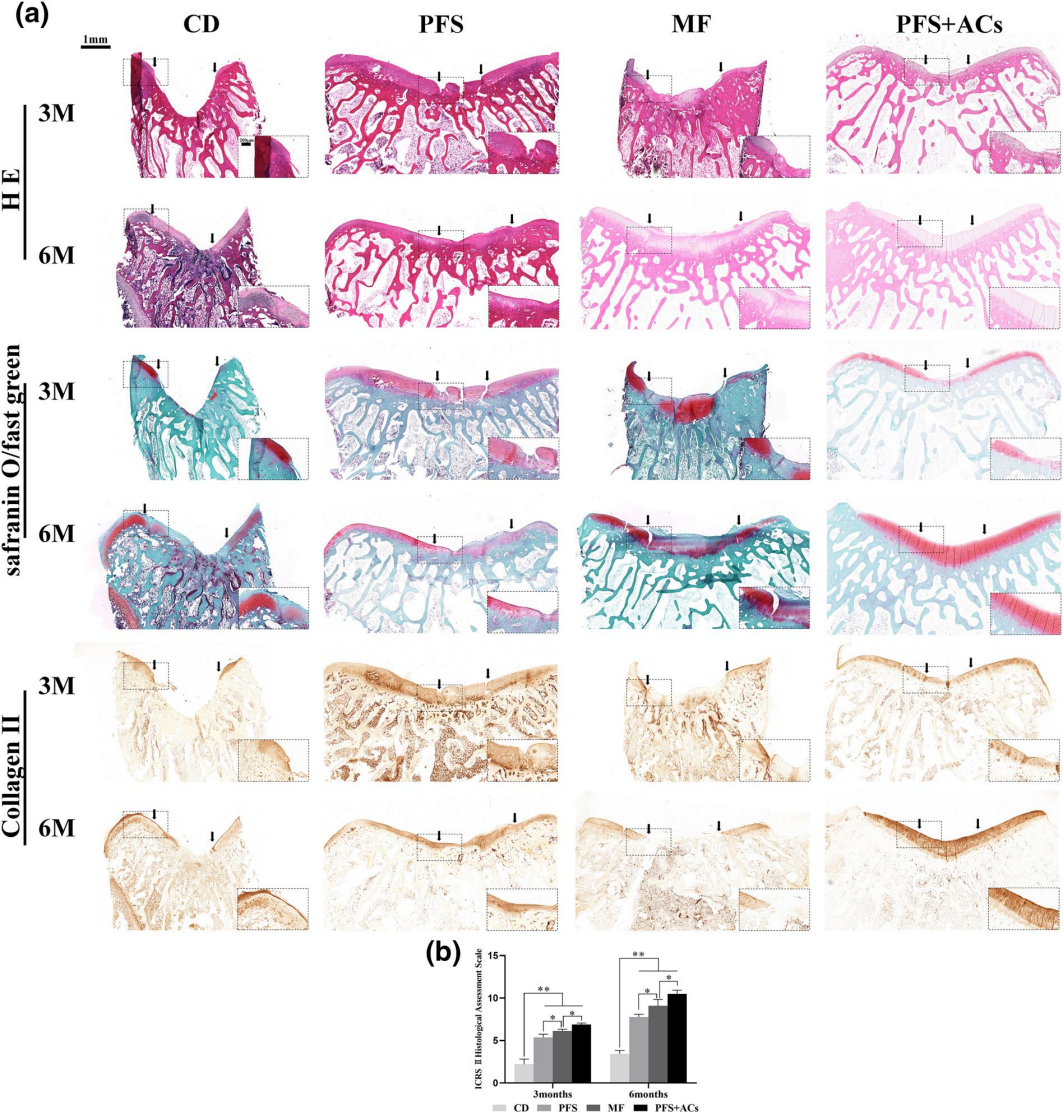
(a) H&E, safranin O/fast green and type II collagen immunohistochemical staining of regenerated cartilage in each group at each time point after the operation. (Black arrows denote the repair interface, bars: 1 mm; Black box indicates a partial magnification, bars: 200 μm). (b) ICRS II Visual Histological Assessment Scale score for each group. The results are presented as the mean ± SD (*n* = 3, *p < 0.05, **p < 0.01) H&E, haematoxylin and eosin; ICRS, international cartilage repair society

Three and 6 months postoperatively, H&E, safranin O/fast green and type II collagen immunohistochemical staining of the repaired cartilage in each group was analysed (Figure 5a). At 3 months, the defect area in the CD group remained concave, without tissue formation and negative GAG and collagen II staining results. The defect region in the PFS group was filled with fibrous tissue with a few disordered fibroblast‐like cells. The GAG and type II collagen staining of the repaired tissue was weaker than the normal cartilage tissue on both sides of the defect, and a large area of deficiency appeared. Extensive fibrous tissue filled the defect area in the MF group, and the boundary between the defect and the normal cartilage tissue was obvious. Many disordered fibroblast‐like cells were found in the new tissue. The staining results in the repaired tissue were uneven compared to the extensive and uniform staining of GAG and type II collagen in the normal cartilage tissue on both sides of the defect, and there were a large number of negative results. However, the thickness of the regenerated tissue was significantly lower than the surrounding normal cartilage tissue, and a large number of columnar and clustered chondrocytes were observed in the regenerated tissue in the PFS + ACs group. The new tissue had a smooth continuity and was well integrated with the surrounding normal tissue. Although the staining results of GAG and type II collagen in the new tissues were weaker than the surrounding tissues, the staining results were uniform and continuous.

Six months after the operation, the defect in the CD group was filled with a small amount of fibrous tissue, which was negative for GAG deposition and type II collagen. The new tissue in the PFS and MF groups contained columnar and clustered chondroid cells, nonuniform GAG staining results, and weak expression of type II collagen. However, the new tissue in the PFS group showed uneven thickness and poor continuity compared to the MF group. Notably, the defect in the PFS + ACs group was completely filled with cartilage‐like tissue, in which a large number of typical chondrocytes arranged in columns similar to normal cartilage was observed. GAG content and type II collagen expression were consistent with the surrounding normal cartilage, and the integration of the new tissue was continuous and smooth.

The results of the ICRS II Visual Histological Assessment Scale showed the best reparative effect in the PFS + ACs group 3 and 6 months after the operation, with a significant difference compared to the other groups (Figure 5b).

## 4 DISCUSSION

Fibrin is a natural nanoscaffold that is used to initiate haemostasis and providing a temporary structure to promote cellular activity and the deposition of new ECM (Shiu et al., 2014). It has extraordinary biocompatibility, biodegradability, inherent biological activity and many other unique properties, which makes it a very distinctive biomaterial for cartilage tissue engineering (Noori et al., 2017).

SEM images showed that a large number of fibres in the PFS were superimposed on and intertwined with each other to form a 3D reticular structure, which yielded a high area/volume ratio. This unique network structure facilitates cell attachment, cell‐matrix interaction, and nutrient or metabolite transport. The fibre thickness, pore diameter and pore depth may be adjusted by changing the concentration of fibrinogen, thrombin and XIII factor (Kurniawan et al., 2014). Kim et al. found that 30 mg/ml fibrinogen and 100 IU/ml thrombin were the best conditions for chondrogenesis. Our study used 30 mg/ml fibrinogen to fill cartilage defects, which is consistent with the Kim et al. study. To make PFS fit better with the shape of the cartilage defect in a liquid state and control the timing of PFS polymerization into gel, we determined that 42 IU/ml thrombin was ideal and had little effect on cell morphology. Lower thrombin activity provides a more suitable environment for cartilage repair (Irwin et al., 2019). Moreover, the change in thrombin concentration had little effect on cell morphology (Kim et al., 2020).

As a scaffold material, PFS provides temporary ECM, which is conducive to cell proliferation. The results of the Transwell assay showed that chondrocytes migrated into PFS. The reparative effect on rabbit cartilage defects was significantly better in the groups with PFS than the CD group, which suggests that PFS has a certain recruitment effect on chondrocytes and other sources of marrow stromal cells (MSCs) in the knee joint. Fibrinogen promotes the proliferation and gene expression of MSCs, which promote the regeneration of cartilage (Lee et al., 2012). Chondrocytes grow from shredded cartilage fragments and migrate into FS (Levinson et al., 2019). However, the repair effects of the PFS group were worse than the MF and PFS + ACS groups, which indicates that FS had poor ability to recruit endogenous chondrocytes and stem cells, and further supplementation with exogenous cells was needed.

The fibrin scaffold is an excellent cell carrier. PFS and chondrocytes were co‐cultured in vitro for 0, 7 and 14 days and observed using confocal microscopy. The chondrocytes were uniformly distributed in the PFS scaffold and exhibited good growth state and activity. With the extension of culture time, there was fusion between the cells. The CCK‐8 proliferation assay also confirmed that PFS promoted the proliferation of ACs. Buchberger et al. performed 3D culture of chondrocytes and a fibrin‐thrombin mixture in vitro and confirmed that the mixture promoted chondrocytes proliferation and cartilage formation (Stefanie Buchberger et al., 2020). Yue et al. observed that the chondrocytes/chondrocyte‐microtissue mixture was well distributed on an ear scaffold of FS. The cartilage matrix secreted by this mixture gradually replaced the FS to form a solid auricle structure (Yue et al., 2021). Three‐dimensional culture in FS did not affect the phenotype of ACs (Bianchi et al., 2019).

Preparing a mixed cell‐fibrin hydrogel prevents cell leakage, and the hydrogel does not completely harden in a short time. This approach ensures that the initially expected hydrogel volume stays in the defect and perfectly integrates with the surrounding tissue according to the shape and size of the defect. During the process of polymerization, the gel achieves a uniform cell distribution via the process of rapid immobilization. FS enables mechanical adhesion, and it is beneficial for the biological integration of implants. FS scaffolds carry cells and promote the formation of host‐scaffold interfacial tissue (Drobnic et al., 2018). No inflammatory reaction was observed in any of the repaired sites 3 or 6 months after the operation in the PFS and PFS + ACs groups, which supports the perfect biocompatibility of the xenogeneic PFS in the rabbit knee joint. We did not use an additional graft fixation technique to create the defect, even in these highly mobile experimental animals, and PFS maintained its stability in situ, which confirmed its excellent adhesion performance and ability to adapt to cartilage defects. Barros et al. reported that a similar FS derived from snake venom had sufficient viscosity, stability, and no adverse reactions in animal experiments and promoted cartilage repair (de Barros et al., 2016).

The new cartilage formed by fibrin scaffolds has histological characteristics similar to natural cartilage (Cakmak et al., 2013). The mechanical properties of cartilage tissue largely depend on the composition and structure of the ECM (primarily type II collagen and proteoglycan; Ahmed et al., 2011). Type II collagen provides the strength for cartilage to resist tension, and proteoglycan allows cartilage to resist compression (Vinatier et al., 2009). Our animal experimental results showed that the contents of total collagen and GAG were highest in the PFS + ACs group, and the safranin O/fast green and immunohistochemical type II collagen staining results were the strongest. These results indicate that the regenerated cartilage in this group contained the most type II collagen and proteoglycan, and its mechanical properties should be the best, which was confirmed in the biomechanical testing. Although the mechanical properties of PFS are poor, the mechanical properties of the regenerated cartilage in the PFS + ACs groups were significantly better than the traditional MF treatment group. Khanmohammadi et al. wrapped menstrual blood‐derived stem cells in FS to repair osteochondral defects of the rabbit knee joint, and the regenerated cartilage tissue was superior to FS alone in the content of GAG and type II collagen and integration (Khanmohammadi et al., 2019). Choi also used allogeneic chondrocytes and autologous bone marrow cells in combination with fibrin to repair full‐thickness cartilage defects in rabbits, and good quality hyaline cartilage was produced (Choi et al., 2018). Almeida et al. used granular cartilage ECM combined with functionalized fibrin hydrogel to form excellent cartilage tissue in vivo and in vitro (Almeida et al., 2016).

The 3D reticular structure of FS has no specific orientation in its natural state. After the application of continuous strain to the viscoelastic FS, it showed a fibre bundle structure that was consistent with the strain direction (Matsumoto et al., 2007). Cell proliferation and matrix mineral deposition in strained FS are also limited in the same direction. The pathological results in the PFS + ACs group in our experiment showed that the vertical arrangement of chondrocytes in the regenerated tissue was consistent with the normal cartilage tissue.

However, PFS alone is limited by rapid degradation and poor mechanical properties, and it cannot provide all of the properties needed for cartilage tissue engineering scaffolds. PFS may be properly processed, chemically modified or combined with other polymers (organic/inorganic nanomaterials) and biomolecules (growth factors) via various processing or synthesis methods or a variety of cross‐linking methods to obtain the desired properties.

PFS may be injected with minimally invasive implantation in clinical treatment, which shortens the healing time and reduces patient discomfort, postoperative complications, and medical costs. Although PFS has some disadvantages, such as poor mechanical properties and fast degradation, these problems may be solved with the development of fibrinogen and thrombin as recombinant proteins and the application of biomolecules binding to fibrin nanomaterials (Noori et al., 2017). PFS will be more widely used in the fields of tissue regeneration in the near future.

In conclusion, PFS has a wide range of sources, low production cost, low risk of disease transmission, unique 3D network structure, good biocompatibility, and a controllable degradation process. It also provides a suitable ECM microenvironment for chondrocytes and promotes chondrocyte adhesion and proliferation. PFS may be perfectly matched with cartilage defects during surgery using a minimally invasive injection, and the regenerated hyaline cartilage tissue is similar to natural cartilage tissue.

## CONFLICT OF INTEREST

All authors declare that there are no conflicts of interest regarding the publication of this paper.

## AUTHOR CONTRIBUTIONS

Yu Yang designed and performed the experiments and wrote the manuscript. Xin Wang performed and designed the experiments. Kangkang Zha and Zhuang Tian performed the animal experiments and measured data. Shuyun Liu and Xiang Sui provided instruction on the experimental methods. Zhigang Wang supervised the experiments. Jilian Zheng and Jun Wang performed the in vitro experiments. Xiaobin Tian, Quanyi Guo and Jinmin Zhao funded and conceived the projects and reviewed the manuscript.

## Data Availability

Research data are not shared.
